# Renal function, uraemia and early arteriovenous fistula failure

**DOI:** 10.1186/1471-2369-15-179

**Published:** 2014-11-17

**Authors:** Emma Aitken, Andrew Jackson, Chia Kong, Paul Coats, David Kingsmore

**Affiliations:** Department of Renal Surgery, Western Infirmary, Dumbarton Road, G11 6NY Glasgow, UK; Strathclyde Institute of Pharmacy and Biomedical Sciences, University of Strathclyde, Glasgow, UK; University of Glasgow Medical School, Glasgow University, Glasgow, UK

**Keywords:** Arteriovenous fistula, Uraemia, Renal function, Maturation, Vascular smooth muscle cells

## Abstract

**Background:**

Guidance varies regarding the optimal timing of arteriovenous fistula (AVF) creation. The aim of this study was to evaluate the association between uraemia, haemodialysis and early AVF failure.

**Methods:**

Immunoblotting and cell proliferation assays were performed on vascular smooth muscle cells (VSM) cells isolated from long saphenous vein samples to evaluate the cells’ ability to proliferate when stimulated with uraemic (post-dialysis) and hyperuraemic (pre-dialysis) serum. Clinical data was collected prospectively for 569 consecutive radiocephalic (RCF) and brachiocephalic (BCF) fistulae. The primary outcome was AVF failure at 6 weeks. Dialysis status (haemodialysis (HD); pre-dialysis (Pre-D)), eGFR and serum urea were evaluated to determine if they affected early AVF failure.

**Results:**

Human VSM cells demonstrated increased capacity to proliferate when stimulated with hyperuraemic serum. There was no significant difference in early failure rate of either RCF or BCF depending on dialysis status (pre-D RCF 31.4% (n = 188); pre-D BCF 22.4% (n = 165); HD RCF 29.3% (n = 99); HD BCF 25.9% (n = 116); p = 0.34). There was no difference in mean eGFR between those patients with early AVF failure and those without (11.2+/-0.2 ml/min/1.73 m^2^ vs. 11.6+/-0.4 ml/min/1.73 m^2^; p = 0.47). Uraemia was associated with early AVF failure (serum urea: 35.0+/-0.7 mg/dl vs. 26.6+/-0.3 mg/dl (p < 0.001)).

**Conclusions:**

We present the first in vivo evidence of an association between adverse early AVF outcomes and uraemia. This is supported mechanistically by in vitro work demonstrating a pro-mitogenic effect of hyperuraemic serum. We hypothesise that uraemia-driven upregulation of VSM cell proliferation at the site of surgical insult in contributes to higher early AVF failure rates.

## Background

Arteriovenous fistulae (AVF) are the dialysis access modality of choice for patients with end stage renal disease (ESRD) [1, 2]. They are associated with a six-fold reduction in the risk of systemic sepsis [3] and lower all cause and cardiovascular mortality than tunnelled central venous catheters (TCVCs) [4, 5]. For this reason both the UK Renal Association and the Fistula First Initiative in the United States have set targets that two-thirds of incident haemodialysis (HD) patients should commence dialysis via an AVF [5, 6].

There is little evidence however regarding the optimal timing of vascular access creation in incident HD patients. Broad consensus exists that timely surgical referral for access creation is important, however clinical practice guidelines are largely opinion-based and vary widely. Furthermore, the exact timing of dialysis initiation for an individual patient can be unpredictable [7]. The UK Renal Association advocates that referral for vascular access should occur when the patient enters CKD stage 4 taking into account co-morbidities, rate of decline in renal function and the surgical pathway [5]. Similarly, the Canadian Society of Nephrology guidelines recommend that a patient be referred with “creatinine clearance 15-20 ml/min or serum creatinine 3.4-5.6 mg/dl (300-500 μmol/l) depending on the size and weight of the patient” [8]. In contrast, the 2006 Kidney Disease Outcomes Quality Initiative (KDOQI) guidelines provide a timeframe for referral “at least six months before the anticipated need for dialysis” [9].

One of the major problems in vascular access planning is the unpredictability of successful maturation. Initial patency rates vary from 60-80% [10, 11] and one recent multicentre study found that 60% of AVFs were not suitable for cannulation 4-5 months after creation [12]. Various risk factors for early AVF failure have been identified including advancing age [13], female gender [14] and diabetes [15].

Several authors have speculated that the timing of access creation itself may influence AVF outcome. Data from the Dialysis Outcomes and Practice Patterns Study (DOPPS) indicates that patients are less likely to start HD via an AVF if there was a longer time from referral to surgical evaluation or longer time from creation to first cannulation [16]. The presence of a central venous catheter at the time of commencing dialysis has also been shown to be associated with poor AVF maturation [17]. Similarly, Weber and colleagues demonstrated a trend towards improved patency in AVF created when the patient was pre-dialysis, but the study was underpowered to formally assess this secondary outcome measure [18].

Associations between the uraemia which occurs in chronic kidney disease (CKD), altered immune response [19] and deranged vascular biology [20, 21] are well recognised. It may be, therefore, that the uraemic state of patients with ESRD influences AVF outcomes. In a rat model of AVF maturation, Langer and colleagues found inferior vessel dilatation and an exacerbation of neointimal hyperplasia in uraemic animals [22]. These factors may need to be taken into account when planning and timing AVF creation.

The aim of this study was to evaluate the hypothesis that uraemia drives vascular smooth muscle (VSM) cell proliferation and impairs AVF maturation. In-vitro studies using VSM cells exposed to serum sampled from subjects prior to a dialysis session (pre-HD) and after a dialysis session (post-HD) were undertaken to quantify the direct effect of hyperuraemic serum on cell growth. Early fistula failure rates (6 weeks) were then compared between pre-dialysis patients and those already on haemodialysis for different eGFR and serum urea at time of AVF creation and (in those patients who had already commenced HD) between patients who dialysed on the same day as surgery and those who dialysed on the day prior to surgery.

## Methods

Approval was obtained from the West of Scotland Research Ethics Committee. Research was conducted in accordance with the Declaration of Helsinki. All subjects providing blood samples provided written informed consent.

### In vivo clinical study

Retrospective analysis of a prospectively collected database of all simple arteriovenous fistulae created in our tertiary referral vascular access centre during a three-year period (January 2010-December 2012) was performed. Patients were excluded if they were switching from peritoneal dialysis to HD or if they had a failing transplant. Patients undergoing brachiobasilic AVF creation were also excluded, so the study population only included brachiocephalic (BCF) and radiocephalic (RCF) fistulae. Any patient who lost their AVF within six weeks for reasons other than thrombosis i.e. ligation for steal or bleeding was also excluded.

Demographic details (age, sex, number of previous fistulae), operative details (site of AVF, type of anaesthetic) and details regarding dialysis status and renal function (dialysis modality, estimated glomerular filtration rate (eGFR) and serum urea in patients who were pre-dialysis (Pre-D) at the time of AVF creation and whether or not the patient had pre-operative haemodialysis on the day of surgery in patients who were already dialysis dependant (HD)) were recorded. Serum urea and eGFR results were obtained within 2 weeks prior to surgery. eGFR was calculated using the Modification of Diet in Renal Disease 4-variable (MDRD-4) formula.

The primary outcome variable was clinical patency at 6 weeks. Clinical patency was defined as an AVF with thrill and bruit and adequate maturation to permit needle cannulation if required as assessed by Vascular Access Specialist Nurses. Secondary outcomes were functional patency (defined as the ability of the AVF to sustain six consecutive dialysis sessions with two needles in those patients who required haemodialysis), clinical patency at time of hospital discharge (defined as the presence of thrill or bruit) and date of loss of clinical patency.

Statistical analysis was performed using the Statistical Package for Social Sciences (SPSS) version 19.0 (SPSS, Chicago, IL). Patients were stratified according to site of AVF. Dialysis status (HD or pre-D), eGFR, serum urea and whether or not the patient dialysed pre-operatively were evaluated to determine if they affected early AVF failure. Normality of continuous variables was assessed using the Shapiro-Wilk test and normal distribution confirmed. Results are presented as a mean ± SEM or percentage of the total population. Continuous data were compared using a Mann Whitney U-test and categorical data compared using chi-squared test. Risk factors for AVF failure were evaluated using logistic regression analysis. Linearity, normal distribution and constant variance of residuals were confirmed to demonstrate no significant interaction or relationship between variables. Variables that were not statistically significant were removed for the model sequentially until the strongest model was obtained. Odds ratio (OR) and 95% confidence intervals are presented. Only patients who had complete demographic details available were considered for multivariate analysis. Kaplan Meier survival curves were used to assess long-term patency of both RCF and BCF. These were compared using a log-rank method. p < 0.05 is considered significant.

### In vitro study

#### Western blotting

Immunoblotting was undertaken as previously described [23]. VSM cells isolated from a section of long saphenous vein were seeded into six well multi plates and grown to ~80% confluence. The medium was then replaced with 0.1% serum for 36 hours. Hyperuraemic pre-dialysis (pre-HD) or less uraemic post-dialysis (post-HD) serum (15% v/v) was added for 15 minutes to stimulate mitogenic signalling pathways. Cells were homogenised in cell lysis buffer and total protein was measured using Bio-Rad protein assay from a 10 μl aliquot of the homogenate (absorbance measured at 595 nm). Samples were then diluted to a total protein content of 1 μg/μl. A total of 20 μg of protein was added to each lane for electrophoresis and transferred to a nitrocellulose membrane (Bio-Rad, Protean 2 system). Immunoblotting was undertaken using antibodies for total ERK and phosphorylated ERK (Santa Cruiz Biotechnology; sc-292838, sc-7976). After incubation with appropriate secondary antibodies, blots were incubated in enhanced chemiluminescence reagents (Amersham, UK) and exposed to photographic film (Kodak X-OmatLS) to detect protein expression. The reactive bands were analysed quantitatively by optical densitometry using a GS-800 imaging densitometer (Bio-Rad), and the amount phosphorylated ERK expressed as a ratio of total ERK.

#### Cell proliferation: 3H thymidine assay

Cell proliferation was determined using serum-induced [3H] thymidine incorporation as described previously [23]. Briefly, VSM cells were seeded at 20,000 cells/well into a 24 well plate. Cells were then quiesced for 48 hours in media containing 0.1% (v/v) serum. The quiesced cells were stimulated with serum for 24 hours with the addition of 1 μCi/well of 3H thymidine (Amersham, UK) for the final 6 hours. Thereafter cells were washed three times for 10 minutes with ice cold phosphate buffered saline containing 10% trichloroacetic acid, and finally the contents of each well solubilised with 200 μls lauryl sulphate (10%) plus sodium hydroxide (0.2 M). The solubilised contents of each well were then transferred to scintillant tube containing 2 mls scintillation fluid. Thereafter radioactivity was quantified by liquid scintillation counting of DPM (disintegrations per minute).

## Results

### In vivo clinical study

A total of 705 AVF were created during the three-year period. 12 (1.7%) were excluded as the patient had a failing transplant and 23 (3.3%) were excluded as the patient was on peritoneal dialysis at the time of AVF creation. 102 patients undergoing BBF formation were also excluded, leaving 569 AVF for analysis (287 RCF, 282 BCF). Of these, 216 (38.0%) were created in patients already on haemodialysis and 353 (62.0%) were created in pre-D patients. Complete data was available in 700 patients. Missing data points related to demographic data and were excluded from analysis. Table 1 outlines the patient demographics and operative details.

**Table 1:** **Patient demographics**

	Total population	Patent at 6 weeks (n = 413)	Not patent at 6 weeks (n = 156)	p-value
**Age (years)**	60.5 ± 0.9	53.3 ± 2.2	64.1 ± 2.4	<0.01
**Sex (% age male)**	56.2% (n = 320)	58.3% (n = 241)	50.6% (n = 79)	<0.05
**Previous attempted AVF?**	31.8% (n = 181)	20.5% (n = 85)	61.5% (n = 96)	<0.001
**Anaesthesia**				<0.001
Local	24.1% (n = 137)	16.7% (n = 69)	43.5% (n = 68)	
Regional	50.3% (n = 286)	58.1% (n = 240)	29.5% (n = 46)	
General	25.7% (n = 146)	25.2% (n = 104)	26.9% (n = 42)	

Results are presented as mean ± SEM or percentage of total for the entire population and in patients with achieving AVF patency at 6 weeks and not achieving 6 week patency. P-values compare patent vs not patent populations.

There was no significant difference in the primary outcome (loss of clinical patency at 6 weeks) of either RCF or BCF depending on dialysis status (pre-D RCF 31.4% (n = 188) vs. HD RCF 29.3% (n = 99), p = 0.34; pre-D BCF 22.4% (n = 165) vs. HD BCF 25.9% (n = 116), p = 0.43) (Table 2). There was no significant difference in either patency on discharge or functional patency between pre-D and HD groups (Table 2).

**Table 2:** **Early patency for Pre-D and HD patients**

	Radiocephalic		p-value	Brachiocephalic		p-value
	Pre-D	HD		Pre-D	HD	
**Primary outcome**						0.43
Clinical patency at 6 weeks	69.6% (n = 130)	71.7% (n = 71)	0.34	77.6% (n = 128)	74.1% (n = 86)	
**Secondary outcomes**						0.72
Patency on discharge	90.4% (n = 170)	91.9% (n = 91)	0.76	89.1% (n = 147)	90.5% (n = 105)	
Functional patency*	80.3% (n = 98)	80.2% (n = 65)	0.87	84.1% (n = 122)	81.7% (n = 85)	0.79

Comparison of AVF outcomes of RCF and BCF created in patients who were pre-HD and those on HD at the time of AVF creation. *Functional patency is the ability of an AVF to sustain HD for 6 consecutive sessions with two needles at any time during the follow-up period. AVF which failed to achieve initial patency on discharge and AVF which never required needling (i.e. the patient remained pre-D or was transplanted prior to ever using the AVF) were excluded from this analysis.

There was no difference in mean eGFR between those patients with early AVF failure (loss of clinical patency at 6 weeks) and those without (11.2+/-0.2 ml/min/1.73 m^2^ vs. 11.6+/-0.4 ml/min/1.73 m^2^; p = 0.47) (Table 3). Uraemia was strongly associated with loss of clinical patency at 6 weeks. Mean serum urea in pre-D patients with early AVF failure was 35.0+/-0.7 mg/dl compared to 26.6+/-0.3 mg/dl in those with patent AVF at 6 weeks (p < 0.001). Similarly, in patients already established on HD, loss of clinical patency at 6 weeks was more likely to occur in patients who dialysed the day prior to surgery for AVF creation compared to those who dialysed on the same day as AVF creation (32.9% vs. 17.7%; p = 0.005) (Table 3). The logistic regression model is shown in Table 4. Uraemia, age, sex and site of AVF were independently associated with AVF patency at 6 weeks. eGFR was removed from the model as there was an relationship with uraemia and dialysis status removed as there was no statistically significant relationship.

**Figure 1:**
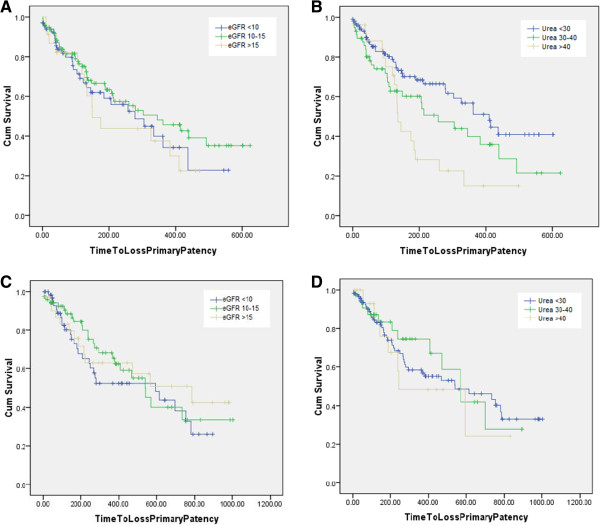
**Kaplan Meier survival curves comparing long term AVF clinical patency stratified by eGFR (<10 ml/min/1.73 m** ^**2**^ **; 10-15 ml/min/1.73 m** ^**2**^ **; >15 ml/min/1.73 m** ^**2**^ **) and serum urea (<30 mg/dl; 30-40 mg/dl; >40 mg/dl) in patients who were pre-D at the time of AVF creation. A**. There was no difference in long-term RCF patency with different eGFR (p = 0.38). **B**. Long-term patency of RCF was better in patients with lower serum urea at the time of AVF creation (p = 0.01). **C**. There was no difference in long-term BCF patency with different eGFR (p = 0.61). **D**. There was no difference in long-term BCF patency depending on serum urea at time of AVF creation (p = 0.79).

Long-term clinical patency of RCF was better in patients with lower serum urea when the AVF was created (p = 0.01; Figure 1B). This association between uraemia and AVF failure was not seen in BCF (p = 0.78; Figure 1D). Similarly there was no association between eGFR and long term RCF (p = 0.38; Figure 1A) and BCF (p = 0.61; Figure 1C) patency.

**Table 3:** **The effect of eGFR, uraemia, and pre-operative haemodialysis on early AVF patency**

	Clinical patency at 6 weeks	Failure to achieve clinical patency at 6 weeks	p-value
**eGFR (ml/min/1.73 m** ^**2**^ )*	11.6 ± 0.4	11.2 ± 0.2	0.47
Serum urea (mg/dl)*	26.6 ± 0.3	35.0 ± 0.7	<0.001
Pre-operative HD**			0.005
Percentage of patients having pre-operative HD	82.3%	17.7%	
Percentage of patients not having pre-operative HD	67.1%	32.9%	

Effect of eGFR, uraemia, and pre-operative haemodialysis on early AVF failure (loss of clinical patency at 6 weeks). Results are presented as mean+/-SEM *For patients who are pre-D; **For patients who are already dialysis dependant at time of AVF creation.

**Table 4:** **Logistic regression analysis for factors associated with early AVF failure**

Coefficient	Estimate	Odds ratio	95% CI	p-value
**Intercept**	60.5			
**Age (adjusted)**	0.67	1.21	1.12,1.33	<0.01
**Sex**	0.65	1.32	1.06,1.58	<0.01
**Site of AVF**	0.06	2.40	1.25, 3.55	<0.001
**Serum urea**	0.04	1.6	1.06,2.14	<0.05

Logistic regression analysis evaluating risk factors for early AVF failure. Age, sex, site of AVF and serum urea (OR 1.6) were found to be independently predictive of AVF failure at 6 weeks.

### In vitro study

**Figure 2:**
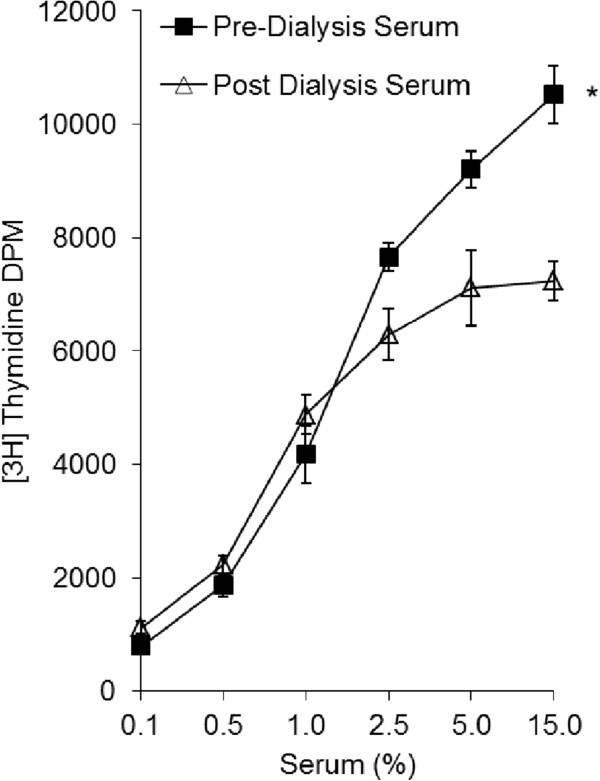
**The effect of human serum pre and post-dialysis on VSM cell proliferation.** *p < 0.05 one way ANOVA for repeated measures, post dialysis serum vs. pre dialysis serum, n = 6 for each.

**Figure 3:**
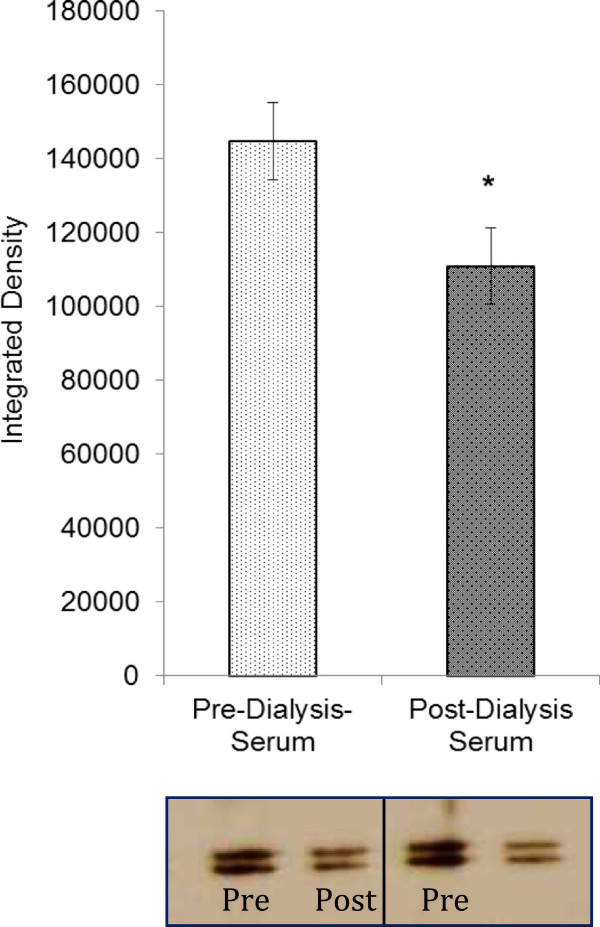
**The effect of 15% human serum pre and post-dialysis on phosphorylation of ERK 1/2 in VSM cells.** *p < 0.05 unpaired, two tailed t-test, post dialysis serum vs. pre dialysis serum, n = 5 for each. Insert below shows two examples of blots where pERK 1/2 are measured following stimulation with 15% serum taken either pre or post dialysis.

Human VSM cells demonstrated increased capacity to proliferate when stimulated with hyperuraemic serum obtained immediately prior to haemodialysis (pre-HD) when compared with cells stimulated with serum following dialysis (post-HD) (Figure 2). Both the sensitivity to serum and the maximum stimulatory response was significantly increased in the pre-dialysis serum when compared to the post-dialysis serum. The maximum stimulatory growth response of VSM cells to 15% pre-dialysis serum was 1.47 ± 0.2 times that of the post-dialysis serum (p < 0.05). Figure 3 demonstrates the effect of 15% serum on stimulation of the mitogenic ERK 1/2 VSM cell signalling pathway. Pre-dialysis hyperuraemic serum significantly increased phosphorylation of ERK 1/2 in VSM cells when compared with post-dialysis serum by a factor of 1.37 ± 0.1 (p < 0.05).

## Discussion

We report the outcomes of a prospective, single-centre observational study comparing early AVF failure rates in patients who were pre-dialysis (pre-D) at the time of AVF creation and those already on haemodialysis (HD). Uniquely, these results are supported by basic science work from our own group, providing mechanistic explanation and validity for the results observed.

We found no difference in the primary outcome, patency at 6 weeks, between pre-D and HD patients for either BCF or RCF. This novel finding is in conflict with the results of Rayner and colleagues, who concluded that dialysis via a TCVC at the time of access creation was associated with early access failure [24]. Similarly, Weber and colleagues achieved excellent “real world” outcomes in their cohort of patients who had AVF created prior to starting HD compared their cohort of patients who had AVF creation delayed until after commencing HD, with 81% vs. 44% of patients using an AVF at 6 months [18]. It should be noted however that the criteria for referral for access creation in this study was an eGFR ≤25 ml/min, whilst the mean eGFR at time of AVF creation in our study was 11 ml/min.

This is the first clinical study to evaluate the relationship between renal function and AVF outcome. We found no association between eGFR at the time of access creation and either short or long-term patency. However increasing serum urea was associated with worse clinical patency at 6 weeks and poorer long-term outcomes from RCF. Similarly, in those patients who had already commenced HD at the time of access creation, dialysis on the same day as surgery was associated with better early patency rates. These findings are consistent with our cell signalling experiments. We hypothesise that the VSM cell proliferation and neointimal hyperplasia which occur at the site of endothelial injury and surgical trauma when the AVF is created are exacerbated by the pro-mitogenic effect of uraemic serum and are deleterious to early fistula maturation.

The factors affecting AVF maturation are multifactorial including vascular anatomy [25] and haemodynamics [26, 27], vessel quality [28] and immune and biochemical properties [20, 21]. Fistulae require adequate arterial inflow and venous outflow to permit maturation. Inflow may be compromised by technical failure at the anastomosis or a poor quality arterial tree. Outflow may be impaired by anatomical or technical “kinking” of the vessels or altered vascular biology leading to VSM cell proliferation, neointimal hyperplasia and venous outflow stenosis. Uraemia alters vascular biology, physiology and biochemistry [19–21] and may contribute to both inflow and outflow difficulties.

Both arterial stiffness and vascular calcification are increased in uraemic patients. In particular, calcification of the media is unique to ESRD and may impair AVF maturation by limiting arterial inflow [29]. Atherosclerosis is accelerated in patients with chronic kidney disease (CKD) with increased cardiovascular mortality [30] and increased intima-medial thickness in both coronary and carotid arteries [31, 32]. An increased intima-medial thickness is seen in the radial artery of uraemic patients and is associated with poor arterial inflow and failure of maturation in RCF [33]. Most studies of CKD-mediated vasculopathy focus on the arterial system, however it’s likely that the detrimental effects of uraemia also affect the venous system in a similar manner [34]. Arterial calcification is well known to impair an artery’s ability to distend upon high flow stimulation [35]. Lee and colleagues have recently demonstrated extensive calcification within the intima and media of venous segments harvested at the time of vascular access surgery which, similar to in the arterial setting, may result in reduced venous compliance and inhibition of the outward remodelling of the venous outflow required for AVF maturation [34, 36].

VSM cell proliferation and neointimal hyperplasia occur at the sites of vessel injury, for example the surgical anastomosis, leading to perianastomotic stenosis [37]. Uraemia has previously been shown to promote neointimal hyperplasia, inhibit vascular repair and promote stenosis in a rodent fistula model [22]. Additionally endothelial progenitor cells (EPCs), which contribute to vessel repair and neovascularisation, have reduced ability to migrate in uraemic serum [38]. In the present study we have, for the first time, isolated human VSM cells and exposed them to hyperuraemic serum and compared proliferation and the associated pro-mitogenic signalling. Our observations support the notion that hyperuraemic serum contains pro-growth factors which upregulate VSM cell proliferation and neointimal hyperplasia leading to early AVF failure. This process may occur de novo at the site of surgical injury or may be an exacerbation of existing neointimal hyperplasia of the outflow vein, which is known to predate AVF creation in patients with CKD [39]. The clinical consequences are likely to be most marked in small vessels, as evidenced by the poorer long-term outcomes of RCF and not BCF created in uraemic patients.

As with many in ESRD, our study is limited by a heterogenous patient population. Multiple potential confounding variables exist. In particular, our assertion that the improved AVF outcomes in patients who dialyse on the same day as AVF creation reflects reduction in serum urea may be erroneous. There are many potential confounding variables, including the administration of systemic heparin and optimisation of cardiovascular function, which could improve AVF outcomes in patients having HD on the same day as surgery. Secondly, our primary endpoint of clinical patency at six weeks is vulnerable to observer bias with different clinicians interpreting patency differently. We chose this pragmatic endpoint which does not include cannulation in an attempt to permit comparison between pre-D and HD patients. By using experienced Vascular Access Nurse Specialists to assess patency we have attempted to maintain standardisation and perform a clinically relevant assessment of outcome.

Our results indicate the uraemia (independent of dialysis status or eGFR) is a risk factor for early AVF failure. This has significant clinical implications regarding the timing of referral for AVF creation. Whilst most authors favour early referral [8, 40], a recent sensitivity analysis actually suggests comparable life expectancy and improved quality of life for patients with CKD stage 4 when a watchful waiting approach to access creation is adopted [41]. We would support creation of AVF in all incident patients prior to starting HD. Our results indicate that, in order to optimise maturation rates, even earlier referral prior to development of significant uraemic symptoms may be required. Secondly, for a number of years it had been local practice to expedite access creation in patients with rapidly declining renal function and progression to end-stage disease. The results of this study have prompted a change in that practice, given the poor AVF outcomes in uraemic patients. It may be that there is a subset of patients who are imminently requiring haemodialysis who would benefit for commencement of HD via a TCVC, physiological and biochemical optimisation and then AVF creation. Finally, the beneficial effect of same day haemodialysis has significant service provision and logistical implications if it is to be implemented for every patient.

## Conclusions

This is the first clinical study to supporting our own and previously published in vitro and animal models demonstrating the deleterious effects of uraemia on AVF maturation. These findings have clinical implications regarding access planning and optimal timing of referral for vascular access.
